# Neuropsychological and cerebral morphometric aspects of negative symptoms in schizophrenia: negative symptomatology is associated with specific mnestic deficits in schizophrenic patients

**DOI:** 10.1186/s12888-014-0326-4

**Published:** 2014-11-25

**Authors:** Tobias Hornig, Gabi Valerius, Bernd Feige, Emanuel Bubl, Hans M Olbrich, Ludger Tebartz van Elst

**Affiliations:** Department for Psychiatry, Albert-Ludwigs-University, Hauptstr. 5, Freiburg, 79104 Germany

**Keywords:** Schizophrenia, SANS, Verbal memory, Magnetic resonance volumetry

## Abstract

**Background:**

The prevalence of negative symptoms in schizophrenic patients seems to be an important indicator for treatment response and prognosis. Although negative symptoms have often been attributed to frontal lobe anomalies, neuropsychological and anatomical findings do not explicitly support this assumption. Since knowledge about the cerebral correlate of negative symptoms in schizophrenia might have a strong impact on therapeutic and psychopharmacological interventions, we aimed to answer this question by investigating the relationship between negative symptoms, neuropsychological functioning and cerebral volumes in schizophrenic patients.

**Methods:**

Twenty schizophrenic patients and 32 healthy controls were examined using a neuropsychological test battery for the assessment of temporal (mnestic) and frontal (executive) faculties. Volumetric measurements of temporal (hippocampus and amygdala) and frontal (orbitofrontal, dorsolateral prefrontal, and anterior cingulate area) brain areas were performed. Negative symptoms were assessed using the Scale for the Assessment of Negative Symptoms (SANS).

**Results:**

Schizophrenic patients performed worse than healthy controls in tests assessing verbal and visuospatial learning and memory functions and on the Stroop interference task. After dividing the schizophrenic group in patients with high and low SANS scores almost all of these deficits were restricted to the former group. There were no overall group differences regarding cerebral subarea volumes. Overall negative symptoms were significantly correlated with verbal memory functions but not with frontal lobe faculties.

**Conclusions:**

Negative symptoms in schizophrenia could specifically associated with verbal memory deficits.

## Background

In 1985 Crow suggested a dichotomic concept for the diagnosis of schizophrenia by dividing schizophrenic disorders into type I- and type II-schizophrenia according to the prevalence of either positive or negative symptoms [1]. Since schizophrenic patients often show symptoms of both dimensions and the psychopathology tends to change in the course of disease [2], Crow’s approach did not suffice as a diagnosis system.

Nevertheless the concept of negative symptoms in schizophrenia has attracted much attention, since negative symptoms have been identified as important indicators for treatment response and prognosis. Negative symptoms, especially anhedonia and affective flattening, can be used as predictors of long-term outcome [3,4]. Furthermore negative symptomatology in schizophrenia was found to intensify the severity of illness and strongly influence global functioning [5]. Some authors found a specific influence of negative symptoms on vocational (work performance) and social functioning as well as functional skills [6-8]. Furthermore negative symptoms have been associated with social problem solving [9]. Besides, they may also predict response to certain medication. Clozapine responders were identified by a lesser degree of negative symptomatology [10].

The first rating scale for the assessment of negative symptomatology, the “Scale for the Assessment of Negative Symptoms” (SANS), was developed by Andreasen in the early 1980s [11,12]. The SANS became the standard instrument for rating the extent of negative symptoms in schizophrenia. It defines five symptom complexes: affective flattening, impoverishment of language and thinking (alogia), reduced drive (avolition/apathy), the inability to feel happy (anhedonia/asociality) and disturbance of attention.

Negative symptoms might be differentiated from positive symptoms by a distinct pathological process. Traditionally negative symptoms in schizophrenia have been linked to frontal lobe anomalies, mainly due to the frequently described relationship between negative symptoms and hypofrontality while in a resting state or during the processing of cognitive tasks [13-18].

Due to these findings many studies regarding the correlation between negative symptoms and cognitive dysfunctions in schizophrenia have hypothesized and verified an inverse relationship between the extent of negative symptoms and so-called frontal lobe deficits. Inverse correlations were most often found with paradigms assessing logical thinking and categorization, e.g. the Wisconsin Card Sorting Test as well as indicators of verbal fluency [19-23].

Nevertheless inverse correlations with a variety of other cognitive dysfunctions have also been found. Pronounced negative symptoms have been linked to stronger impairments in recognition memory, psychomotor speed, attention or visuospatial abilities [22,24-27].

Several morphometric studies support the link between negative symptoms in schizophrenia and frontal lobe impairment. Reduced frontal lobe volumes have been found in correlation with anhedonia and the apathy level of schizophrenic patients, which are core symptoms of schizophrenic negative symptomatology [28,29]. The extent of negative symptoms was also significantly correlated with smaller orbitofrontal, left insular, dorsolateral prefrontal regions, bilaterally in the medial frontal, anterior cingulate, inferior frontal and superior temporal regions gray matter volume [30-32] as well as reductions in prefrontal gray and white matter [21,33,34]. Asami and coworkers found a positive correlation between frontolimbic and left superior temporal gyrus gray matter volume reduction and negative symptoms [35]. Reduced gray matter volume in the middle prefrontal gyrus (Brodmann area 9/46 of frontal association cortex) as a correlate of negative symptoms have also been shown by other authors [36-38]. Following Talati and Hirsch the medial prefrontal gyrus, appendant to the dorsolateral prefrontal cortex, seems to be associated with executive mechanisms and decision- making abilities as a higher processing unit and might thereby modulate negative symptoms [39]. A study using diffusion tensor imaging, a method to assess the integrity of neural fiber tracks, suggests an association between impaired white matter integrity in the inferior frontal region of schizophrenic patients and the severity of negative symptoms [40]. However, in analogy to the neuropsychological findings, these results do not seem to be unambiguous, since other studies reported correlations between negative symptomatology and anomalies in other brain areas, especially the temporal regions, with the superior temporal gyrus [34,41-43] and the hippocampus being most often reported [44].

So far distinct conclusions concerning the relationship between cognitive dysfunctions and negative symptoms in schizophrenia especially with respect to the cerebral correlate cannot be drawn. Based on present findings anomalies connected to the frontal and temporal lobes are primarily discussed.

A disadvantage of many neuropsychological studies examining the association between negative symptoms and neuropsychological dysfunctions is the restriction to a single cognitive faculty or a group of functions related to only one brain region, especially the frontal lobes. Furthermore only few studies have combined a neuropsychological assessment with a morphometric approach to explore the relationship with negative symptoms.

To address this question this study aims to investigate the cognitive and cerebral correlates of negative symptomatology in schizophrenia.

Schizophrenic patients and healthy controls were examined using the SANS, volumetric MRI-measures of different frontal and temporal brain structures and a comprehensive neuropsychological test battery covering frontal lobe (executive) as well as mediotemporal (memory) functions to assess the relationship between negative symptoms, neuropsychological deficits and structural brain anomalies in patients with schizophrenia.

## Methods

### Participants

Approval from the Albert-Ludwigs university ethics committee Freiburg was obtained before onset of the study. 20 patients with DSM-IV-defined schizophrenia, 8 female, 12 male, hospitalised for acute exacerbation and adjusted to medication, were identified among the in-patients of the Department of Psychiatry and Psychotherapy of the University Hospital of Freiburg. The clinical diagnosis of schizophrenia was made by experienced senior consultant psychiatrists based on a structured interview according to DSM-IV criteria. After giving informed written consent to participate on a voluntary basis, patients were included in this study. All patients received a full neurological and medical history and examination as well as routine blood tests and EEG examinations to exclude any other medical or neurological disorder. Patients were excluded if they had any other DSM-IV axis I diagnosis or met criteria for substance abuse within the previous 6 months. To keep the sample homogenous by avoiding an overlap to psychogeriatric problems the study group was restricted to patients under the age of 45 years and normal IQ range. Clinical state of patients was assessed using the Brief Psychiatric Rating Scale (BPRS) [45] and the Scale for the Assessment of Negative Symptoms (SANS) [11]. The overall severity of psychiatric disturbance for the present state as well as for the past two years was measured using the Global Assessment Scale [46]. The Phillips scale [47] was used to assess the premorbid adjustment of the patients. The history of illness was documented in regard to the age of onset of clinical symptoms, the duration of schizophrenia, the total amount of time hospitalized for schizophrenia and the number of psychotic episodes (Table 1). Confounding variables like family history, any history of meningitis or encephalitis or other brain injuries as well as the present medication were documented. Chlorpromazine-equivalents (CPE) were calculated for the current antipsychotic medication. Due to the vague informations provided by the patients and carers lifetime exposure to antipsychotic medication could not be measured. 32 control subjects, 12 female, 20 male, were recruited via announcements and group matched according to age, sex and school education (Table 1). Exclusion criteria were identical for the patient and control group. In addition, volunteers with a positive personal or family history of any psychiatric disorder were excluded from participation.

**Table 1:** **Demographic data and clinical characteristics of the schizophrenic group with a high SANS score, the schizophrenic group with a low SANS score, and the control group as well as significance of results**

**Demographic data/clinical characteristics**	**Schizophrenics high SANS-score**		**Schizophrenics low SANS-score**		**Controls**		
	**Mean**	**SD**	**Mean**	**SD**	**Mean**	**SD**	**F-value**
Age (in years)	27,90	6,03	30,40	7,78	28,22	5,83	0,53
School education (in years)	12,30	1,25	12,20	1,32	12,13	1,50	0,06
Sex (female/male)	7/3		5/5		22/10		1,30^1^
Age of onset of clinical symptoms (in years)	24,22	3,80	26,10	6,06			0,63
Duration of schizophrenia (in months)	49,78	54,48	58,90	51,31			0,03
Number of psychotic episodes	2,11	2,42	2,00	2,00			0,30
Total amount of time hospitalized for schizophrenia (in weeks)	19,11	19,66	24,80	27,77			0,01
BPRS	45,50	16,86	27,60	8,57			7,36**
Positive family history for psychiatric disorders in 1st and 2nd grade dependant	8		10				4,2^1^
Chlorpromazine-equivalents	10		10				0,8^1^

^1^Pearson’s Chi-Square.

(Spearman rank correlation, * p ≤0,05, ** p ≤0,01).

### Neuropsychological assessment

A comprehensive neuropsychological test battery was administered by a trained and experienced psychologist. The test battery covered the following neuropsychological functions: verbal memory/learning, visual memory/learning, concentration, working memory, verbal fluency, verbal intelligence, interference and abstraction/cognitive flexibility.

Verbal memory/learning were assessed using the subtest Logical Memory, trial 1 and 2, of the revised Wechsler Memory Scale (WMS-R, German edition) [48] and the wordlist of the Nürnberg’s Aging Inventory (Nürnberger Altersinventar, NAI) [49]. The subtest Visual Reproduction, trial 1 and 2, of the WMS-R (German edition) was administered to measure visual memory/learning. Working memory and concentration were assessed using the subtest “digit span” of the Wechsler Adult Intelligence Scale (German edition) [50]. Interference, response inhibition, abstraction and cognitive flexibility were evaluated using the Stroop-Test (German version) [51] and the Wisconsin Card Sorting Test (WCST, pc-edition) [52]. The subtest “Information” of the HAWIE-R [50] was administered to measure verbal intelligence. Verbal fluency was assessed using the verbal fluency test of the Performance Test System [53].

The tests were administered after patients had reached subacute status to avoid confounding psychotic symptoms. The neuropsychological assessment lasted between 1.5 and 2 h.

### Imaging and measurements

Within two weeks to the neuropsychological assessment MRI images were obtained at the Department of Radiology at the University of Freiburg on a 2 T whole-body system (Medspec S200, Bruker BioSpin MRI, Ettlingen - Germany) using a standard quadrature head coil. T1 and T2 weighted coronal images were acquired to screen for brain pathology. For volumetric assessment a T1-weighed 3-D-data set was acquired using a modified driven equilibrium Fourier transfer sequence (MDEFT) [54] with the following parameters: TR =17 ms, TE =5.5 ms, flip angle =30, matrix 256×192×92, FOV =24×23×18 cm^3^. The images were transferred to a UNIX Sun workstation via a network (Sun Microsystems, Mountain View, California, USA). Volumetric measurements of the hippocampus, the amygdala, the dorsolateral prefrontal cortex (DLPFC) and the orbitofrontal cortex (OFC) were performed for both hemispheres by a blinded rater after establishing a good reliability using the interactive software program MRreg [55]. The volumetric assessment followed well established and published protocols by our group [56]. Results of structural imaging as well as functional imaging aspects of parts of the study sample have been published in previous papers [57-61].

### Statistical analysis

#### Demographics

To compare both groups concerning age, education and sex Student’s t-Tests and a Chi Square Test were employed, respectively.

#### Group comparisons – neuropsychology

The neuropsychological test scores were converted to Z-scores (standard equivalents) and single test values covering the same neuropsychological faculty were averaged to define the following factors: verbal learning/memory, visuo-spatial learning/memory, working memory/concentration and frontal lobe functioning. Schizophrenic patients and healthy controls were compared concerning the four different neuropsychological factors using multivariate analyses of covariance (MANCOVA) with CPE as covariate to control for medication effects. Following this post hoc multivariate analyses of covariance were calculated for each single neuropsychological test score, again considering CPE as covariate. In a second step a median split on the SANS score was performed to distinguish schizophrenic patients with high and with low SANS scores. The Median split rendered a clear cut between patients concerning negative symptoms, since the SANS global score in the low score group ranged from 0 to 16 and in the high score group from 24 to 74. The two groups differ significantly in the BPRS Score (Student’s t-Test) so that the median split is also relevant in relation to the BPRS. The statistical procedure explained above was repeated comparing schizophrenic patients with a high SANS score (HSS), schizophrenic patients with a low SANS score (LSS) and healthy controls, using multivariate analyses of variance (MANOVA). Post hoc analysis was done using Bonferroni correction.

#### Group comparisons – volumetric data

The volumes of the hippocampus, the amygdala, the anterior cingulate cortex, the DLPFC and the OC were corrected for differences in total brain volumes following earlier publications by our group [61-64]. The different volumes were also analysed in the two steps described above using Repeated Measurements ANOVA with hemisphere as intra-subject factor and CPE as covariate.

#### Correlation analyses

Correlations between psychopathology, neuropsychological results and volumetric data were computed for schizophrenic patients only using Spearman rank-correlation.

All data were analyzed using SPSS for Windows (Release 11.5). A p-value of p ≤ .05 was chosen as the criterion of significance for all statistical calculations.

## Results

### Patient and control group

The overall group of schizophrenic patients as well as the subgroup of patients with high and low negative symptom scores were matched in terms of age, sex and duration of education. There were no differences between the two schizophrenic subgroups in terms of duration of schizophrenia, age of onset of clinical symptoms, total amount of time hospitalized for schizophrenia and number of psychotic episodes (see Table 1). In relation to the BPRS *schizophrenic patients with a high SANS score differ significantly from patients with a low SANS score. The BPRS scores for the patients with a high SANS score were 45,5 and a BPNRS scores for the patients with low SANS score were 27,6 points (F =7,3, p <0,01).*

### Group comparisons - neuropsychological performance

#### Schizophrenic patients vs. healthy controls

UNIANOVA showed a significantly worse performance of the schizophrenic patients compared to the healthy controls concerning verbal and visuospatial learning/memory (F =9,52, p ≤0,01; F =11,49, p ≤0,01). No differences in performance were found for working memory/concentration and frontal lobe functioning (F =0,28, n.s.; F =2,11, n.s.). Considering the single neuropsychological parameters UNIANOVAs showed a significantly worse performance of the schizophrenic patients compared to the healthy controls concerning WMS-R, Logical Memory immediate recall and delayed recall, NAI Wordlist immediate recall trials 2 and 3 as well as delayed recall, WMS-R, Visual Reproduction immediate and delayed recall and Stroop – Interference. No medication effects were found. Mean raw scores of the neuropsychological parameters for the schizophrenic and the control group as well as significance of results are shown in Table 2.

**Table 2:** **Mean raw scores of the neuropsychological parameters for the schizophrenic and the control group as well as F- and significance of results**

***Neuropsychological test***	***Controls***		***Schizophrenics***		
	**Mean**	**SD**	**Mean**	**SD**	**F-value** ^**1**^
WMS-R, logical memory I	33,28	6,97	39,00	26,10	10,97**
WMS-R, logical memory II	29,63	8,54	37,00	20,40	13,73**
NAI, wordlist trial I	8,22	2,79	7,05	1,76	3,15
NAI, wordlist trial II	11,84	2,48	9,60	2,11	6,33*
NAI, wordlist trial III	13,22	1,75	11,90	1,86	6,30*
NAI, wordlist delayed	11,91	3,38	9,65	3,56	4,09*
HAWIE-R, information	18,94	3,63	15,65	3,84	2,18
WMS-R, visual reproduction I	39,47	1,68	37,75	2,47	7,50**
WMS-R, visual reproduction II	39,13	2,20	36,05	4,76	12,91**
HAWIE-R, digit span	17,25	4,53	15,05	2,96	0,34
HAWIE-R, digit span forward	8,78	2,09	8,20	1,82	0,09
HAWIE-R, digit span backward	8,47	2,85	6,85	1,73	1,34
Stroop interference	63,56	11,97	87,35	23,28	10,23**
Verbal fluency	41,34	15,51	30,40	8,62	3,31
WCST, ntcc1	12,26	3,82	16,28	10,16	0,23
WCST, perseverative reactions	12,39	12,60	19,50	17,52	1,02
WCST, perseverative errors	10,87	10,17	17,33	14,48	1,28

^1^based on the Z-scores of the different neuropsychological test parameters.

WCST, ntcc1 = WCST, no. of trials to complete category 1.

(Spearman rank correlation, * p ≤0,05, ** p ≤0,01).

#### Schizophrenic patients with a high SANS score vs. schizophrenic patients with a low SANS score vs. healthy controls

The MANCOVAS showed significant group effects for verbal and visuospatial learning/memory (F =8,18, p ≤0,01; F =5,67, p ≤0,01). Post-hoc analysis indicated a worse performance of the schizophrenic HSS patients compared to the schizophrenic patients with a LSS and the controls concerning verbal learning/memory (mean difference =0,73, p ≤0,05; mean difference =1,08, p ≤0,01). Concerning visual learning/memory schizophrenic patients with a HSS score achieved lower scores than controls (mean difference =0,78, p ≤0,01). No differences in performance were found for working memory/concentration and frontal lobe functioning (F =0,15, n.s.; F =1,55, n.s.). No medication effects were found.

We found significant group effects for WMS-R, Logical Memory immediate and delayed recall, NAI Wordlist immediate recall trial 2–3 and delayed recall, HAWIE-R, subtest “information” WMS-R, Visual Reproduction immediate and delayed recall, Stroop – Interference, verbal fluency, WCST, no. of trials to complete category 1 and no. of perseverative errors.

Post-hoc analyses indicated a worse performance of the schizophrenic patients with a HSS compared to the controls concerning WMS-R, Logical Memory immediate recall and delayed recall, NAI Wordlist immediate recall, trial 2 and trial 3 and WMS-R, NAI Wordlist delayed recall, HAWIE-R, subtest “information”, WMS-R, Visual Reproduction delayed recall, Stroop – Interference and WCST, no. of trials to complete category 1. Schizophrenic patients with a HSS also achieved lower test scores than schizophrenic patients with a LSS concerning WMS-R, Logical Memory delayed recall. Schizophrenic patients with a LSS showed a worse performance than controls in regard to WMS-R, Visual Reproduction immediate recall, Stroop – Interference and WCST, perseverative errors. The mean raw scores of the neuropsychological tests for the two schizophrenic groups and the control group as well as significance of results are shown in Table 3.

**Table 3:** **Mean raw scores of the neuropsychological parameters for the schizophrenic group with a high SANS score (S1), the schizophrenic group with a low SANS score (S2), and the control group (C) as well as significance of results**

**Neuropsychological test**	**Schizophrenics high SANS-score (S1)**		**Schizophrenics low SANS-score (S2)**		**Controls (C)**			**Post-hoc analyses**		
	**Mean**	**SD**	**Mean**	**SD**	**Mean**	**SD**	**F-value** ^**1**^	S1 vs. S2*	S2 vs. C*	S1 vs. C*
WMS-R, logical memory I	22,80	5,73	29,40	6,15	33,28	6,97	6,58**	n.s.	n.s.	p ≤0,01
WMS-R, logical memory II	15,70	5,98	25,10	5,61	29,63	8,54	8,34**	p ≤0,05	n.s.	p ≤0,01
NAI, wordlist trial I	6,20	1,03	7,90	1,97	8,22	2,79	1,97	n.s.	n.s.	n.s.
NAI, wordlist trial II	8,80	1,93	10,40	2,07	11,84	2,48	4,97**	n.s.	n.s.	p ≤0,01
NAI, wordlist trial III	11,00	1,76	12,80	1,55	13,22	1,75	4,22**	n.s.	n.s.	p ≤0,01
NAI, wordlist delayed	7,90	3,76	11,40	2,41	11,91	3,38	3,7**	n.s.	n.s.	p ≤0,01
HAWIE-R, information	14,80	4,42	16,50	3,17	18,94	3,63	5,5**	n.s.	n.s.	p ≤0,01
WMS-R, visual reproduction I	37,90	2,96	37,60	2,01	39,47	1,68	3,27*	n.s.	p ≤0,05	n.s.
WMS-R, visual reproduction II	34,60	6,29	37,50	1,90	39,13	2,20	5,36**	n.s.	n.s.	p ≤0,01
HAWIE-R, digit span	15,60	3,17	14,50	2,80	17,25	4,53	1,89	n.s.	n.s.	n.s.
HAWIE-R, digit span forward	8,20	2,04	8,20	1,69	8,78	2,09	1,27	n.s.	n.s.	n.s.
HAWIE-R, digit span backward	7,40	1,84	6,30	1,49	8,47	2,85	2,34	n.s.	n.s.	n.s.
Stroop interference	84,00	23,08	90,70	24,22	63,56	11,97	7,85**	n.s.	p ≤0,01	p ≤0,01
Verbal fluency	30,10	9,83	30,70	7,76	41,34	15,51	2,75	n.s.	n.s.	n.s.
WCST, ntcc1	19,60	11,65	12,13	6,36	12,26	3,82	7,07**	n.s.	n.s.	p ≤0,01
WCST, perseverative reactions	14,40	10,16	25,88	23,01	12,39	12,60	2,46	n.s.	n.s.	n.s.
WCST, perseverative errors	13,10	8,50	22,63	18,94	10,88	10,17	2,77*	n.s.	p ≤0,05	n.s.

^1^based on the Z-scores of the different neuropsychological test parameters.

n.s. = not significant.

WCST, ntcc1 = WCST, no. of trials to complete category.

(Spearman rank correlation, * p ≤0,05, ** p ≤0,01).

### Group comparisons – volumetric findings

#### Schizophrenic patients vs. healthy controls

**Figure 1:**
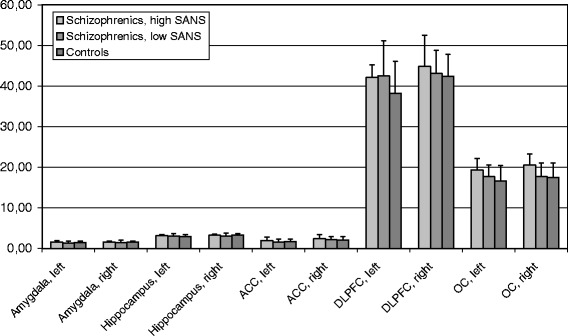
**Mean corrected volumes of the amygdalae, hippocampi, anterior cingulate cortices, DLPFC and OC for the schizophrenic group with a high SANS score, the schizophrenic group with a low SANS score, and the control group [error bar indicates Standard error].**

In 10 control subjects the quality of structural 3-D-data sets was insufficient for further analysis. There were no significant differences between the schizophrenic and the control group with respect to total brain volume (tbv; F =0,89; p = n.s.) as well as the volumes of the hippocampus (F =0,40; p = n.s.), the amygdala (F =0,13; p = n.s.), the anterior cingulate cortex (F =0,00; p = n.s.), the DLPFC (F =0,79; p = n.s.) or the OC (F =0,69; p = n.s.). Neither medication effects (hippocampus: F =0,91; amygdala: F =0,36; anterior cingulate cortex: F =0,39; DLPFC: F =0,15; OC: F =0,33; tbv: F =0,36; p = n.s.) nor any interaction with medication (hippocampus: F =3,11; amygdala: F =2,57; anterior cingulate cortex: F =0,00; DLPFC: F =1,64; OC: F =0,37; p = n.s.) or group (hippocampus: F =0,42; amygdala: F =2,01; anterior cingulate cortex: F =0,07; DLPFC: F =0,01; OC: F =0,05; p = n.s.) were observed. For the hippocampus a significant effect for hemisphere was found (F =15,47; p ≤0,01), indicating a greater right volume of the hippocampus. The same was true for the amygdala (F =5,51; p ≤0,05) and the anterior cingulate cortex (F =4,13; p ≤0,05). An effect for hemisphere was also found for the DLPFC with a greater left than right DLPFC (F =6,81; p ≤0,05). For the OC hemisphere did not show any effects (F =2,16; p = n.s.). The volumetric data for the two schizophrenic groups and the control group are shown in Figure 1.

#### Schizophrenic patients with a high SANS score vs. schizophrenic patients with a low SANS score vs. healthy controls

The UNIANOVA did not show any group effects for total brain volume (F =0,43; p = n.s.). Repeated Measurements ANOVA revealed no group effects (F =0,18; p = n.s.) for the hippocampus. There was a significant effect for hemisphere (F =5,89; p ≤0,05), indicating a greater right volume of the hippocampus, and a significant interaction with group (F =3,37; p ≤0,05). For the anterior cingulate cortex there were no statistically significant effects for group (F =0,56; p = n.s.) or the interaction between group and hemisphere (F =0,12; p = n.s.), but a significant effect for hemisphere corresponding to the lateralization of the hippocampus (F =6,81; p ≤0,05). No group (F =0,54; p = n.s.) or hemisphere (F =2,52; p = n.s.) effects or an interaction between group and hemisphere (F =0,68; p = n.s.) were found for the amygdala. The same was true for the DLPFC (group: F =1,28; p = n.s.; hemisphere: F =3,13; p = n.s.; interaction with group: F =0,71; p = n.s.) and the OC (group: F =2,45; p = n.s.; hemisphere: F =1,55; p = n.s.; interaction with group: F =0,32; p = n.s.). Post hoc analyses did not show any significant differences between any of the three groups in regard to total brain volume, the volume of the hippocampus, the amygdala, the anterior cingulate cortex, the DLPFC and the OC.

#### Correlation analysis

Correlations between SANS sub scores and SANS global score, respectively, and neuropsychological performance are primarily found for indicators of verbal learning/memory. There were no significant correlations between the volumes of the hippocampus, the amygdala, the DLPFC and the OC on the one hand and scores of negative symptoms on the other hand. A positive correlation between total brain volume and the SANS sub score attention was found. Results of correlation analysis can be seen in Table 4.

**Table 4:** **Significant correlations between neuropsychological test results and SANS subscores/SANS global score**

	**Affective flattening**	**Alogia paralogia**	**Abulia apathy**	**Anhedonia associality**	**Attention**	**SANS global score**
WMS-R, logical memory I	−0,33	−0,35	−0,32	−0,49*	−0,38	−0,46*
WMS-R, logical memory II	−0,57**	−0,52*	−0,49*	−0,59**	−0,54*	−0,66**
NAI, wordlist trial I	−0,39	−0,33	−0,46*	−0,29	−0,39	−0,42
NAI, wordlist trial II	−0,48*	−0,57**	−0,25	−0,34	−0,47*	−0,51*
NAI, wordlist trial III	−0,57**	−0,48*	−0,35	−0,34	−0,37	−0,54*
NAI, wordlist delayed	−0,51*	−0,45*	−0,33	−0,38	−0,39	−0,50*
HAWIE-R, information	0,04	0,00	0,04	0,10	−0,18	−0,03
WMS-R, visual reproduction I	0,06	0,15	−0,09	0,00	0,02	0,09
WMS-R, visual reproduction II	−0,34	−0,17	−0,30	−0,15	−0,23	−0,23
HAWIE-R, digit span	0,28	0,03	0,10	0,11	−0,13	0,13
HAWIE-R, digit span forward	0,10	−0,04	0,04	0,12	−0,02	0,05
HAWIE-R, digit span backward	0,42	0,10	0,12	0,01	−0,25	0,17
Stroop interference	−0,07	−0,04	−0,06	−0,19	0,01	−0,07
Verbal fluency	0,03	−0,07	−0,08	−0,30	−0,39	−0,10
WCST, ntcc1	0,05	0,32	−0,03	−0,06	0,03	0,11
WCST, perseverative reactions	−0,04	−0,13	−0,28	−0,26	−0,21	−0,26
WCST, perseverative errors	−0,08	−0,13	−0,29	−0,27	−0,20	−0,28
Total brain volume	0,33	0,31	0,38	0,25	0,63*	0,41
Hippocampus right	0,44	0,24	0,12	0,14	0,10	0,28
Hippocampus left	0,22	0,36	0,10	0,39	0,19	0,26
Amygdala right	0,03	0,18	0,03	0,23	0,31	0,19
Amygdala left	0,12	0,06	0,01	−0,15	−0,02	0,15
Anterior cingulate cortex right	0,02	−0,00	0,23	0,16	0,44	0,24
Anterior cingulate cortex left	−0,11	0,06	−0,15	−0,02	−0,08	0,02
DLPFC right	0,23	0,01	0,26	−0,21	−0,13	0,20
DLPFC left	0,12	0,12	0,10	−0,16	0,09	0,19
OC right	0,36	0,35	0,33	0,24	0,07	0,46
OC left	0,15	0,35	0,33	0,28	0,38	0,43

(Spearman rank correlation, * p ≤0,05, ** p ≤0,01).

## Discussion and conclusions

In this study, schizophrenic patients displayed pronounced impairments in verbal and visuospatial memory as well as deficits concerning response inhibition. Dividing the schizophrenic group in patients with low and high SANS scores demonstrated especially strong memory, but also executive impairments in the HSS patients, whereas the LSS patients were relatively unimpaired. Correlations between SANS sub scores and global score, respectively, were mainly found with indicators of verbal memory. Anomalies in frontal or temporal brain regions or associations with neuropsychological variables were not found. These findings underline the association between negative symptomatology in schizophrenia and pronounced neuropsychological deficits and emphasise the relevance of verbal memory impairments.

### The impact of negative symptoms on neuropsychological impairment in schizophrenia

The neuropsychological findings in our schizophrenic group are in general agreement with findings of other studies showing specific deficits concerning learning and memory in schizophrenic patients [65].

Although the prevalence of executive dysfunctions in schizophrenia is estimated to be around 90% [66] we could not confirm impairments in frontal lobe functioning with the exception of difficulties in response inhibition as assessed by the Stroop task. Results of a study by Hutton [67] indicates, that the type of executive impairment might depend on the phase of illness. At the beginning of the disease process patients display deficits of planning and strategical thinking, whereas in chronic patients this shifts towards impairments concerning categorization and flexibility as assessed by the WCST. Since on average our schizophrenic patients have had only 2 schizophrenic episodes, this might explain the negative WCST results.

Studies have already shown the impact of negative symptomatology on certain neuropsychological functions in schizophrenic patients. Our results confirm the association between negative symptomatology in schizophrenia and pronounced neuropsychological deficits already found by others [22,26,27,29]. Patients with a high SANS global score showed significantly more pronounced deficits especially concerning mnestic functioning than patients with a low SANS global score.

These results underline the close connection between negative symptomatology and verbal memory on the background of macroscopically unchanged structures in frontal and temporal cerebral areas. This is in contrast to other studies, which primarily reported a correlation between negative symptoms and executive functioning, sometimes associated with the corresponding structural changes in frontal lobe regions [19,20,22].

Following the general line of thought a correlation between negative symptoms and executive – or so called frontal lobe - impairments would have been expected, since negative symptoms are assumed to be a consequence of frontal lobe impairment [68]. On the other hand Hill and coworkers observed an amplifying effect of negative symptomatology in combination with disorganized behaviour on learning and memory deficits in schizophrenic patients, thereby confirming the results of this study [69].

The schizophrenic group in this study showed a relatively high level of functioning in the neuropsychological categories studied here. The patients showed almost no executive impairments and no deficits concerning concentration and working memory. Patients with high and low negative symptoms could mainly be separated by their memory performance, even HSS patients could adhere their executive functioning. Memory, especially concerning the verbal modality, should be regarded as left temporal lobe functions in the majority of people and might be more vulnerable in regard to the cerebral changes associated with a schizophrenic disorder than other neuropsychological functions.

Interestingly the schizophrenic patients in this study did neither not show any change in the examined cerebral structures nor any relationship between neuropsychological measures and the examined anatomical faculties. Current findings report contradictory results. Some studies have found smaller volumes of brain structures in schizophrenic patients, especially of the hippocampus, the amygdala and the anterior cingulate cortex [36,70-72]. Whereas other findings could neither find any volumetric differences in the hippocampus-amygdala-complex nor in the different frontal lobe regions [73,74]. Possible explanations for these inconsistencies might be differences in the kind of volume assessment, e.g. morphometric measurements using SPM or manual tracing of a structure according to validated protocols. However, such contradictions could also relate to the fact that the different study samples vary in terms of underlying pathophysiology [75]. Especially in schizophrenia it is extremely difficult to assess a sufficiently homogenous population to be able to generalize findings to the results of other studies. The coexistence of normal volumes of the examined frontal and temporal faculties on the one hand and memory impairments as well as deficits in response inhibition on the other hand allow different interpretations. A functional recovery may have taken place before the structural regeneration. It could also be the case, that the macroscopically normal volume of a certain brain area might mask more subtle histological alterations or functional anomalies not related to structural change.

This leaves the question of the organic basis for the observed deficits and especially the correlation between negative symptomatology and verbal memory impairments unanswered. As macro-structural aberrations do not seem to be the organic basis for the correlation between negative symptoms and verbal memory impairment, biochemical or physiological mechanisms as the underlying common cerebral correlate have to be considered. Glutamate seems to be a good candidate to explain our correlation results. The glutamate hypothesis of schizophrenia was developed after it had been recognized that Phencyclidine (PCP) leads to an exacerbation of symptoms in schizophrenic patients [76] and produces a schizophrenia-like state in normal individuals with positive and negative symptoms as well as cognitive disturbances [77,78]. PCP serves as a noncompetitive antagonist at the glutamatergic NMDA receptor [79]. Glutamate acts via different receptor types, but so far the main focus of the relationship between glutamate and schizophrenia has been on the NMDA receptor. It plays a major role in long-term potentiation, a synaptical process involved in memory formation [80]. Besides its role concerning mnestic functions glutamate also affects schizophrenic negative symptomatology. Using D-serine, a full agonist at the glycine modulatory site of the NMDA receptor, Tsai et al. (1998) have found significant improvements in negative symptoms, psychosis, and executive function as measured by the cognitive subscale of the Positive and Negative Syndrome Scale (PANSS) and performance on the Wisconsin Card Sorting Test [81]. Similarly D-cycloserine, a partial agonist at the glycine modulatory site of the NMDA receptor, has led to a diminution of negative symptomatology either alone or added to conventional antipsychotics [82,83]. Considering these results glutamate might be the latent variable underlying the relationship between mnestic deficits and negative symptomatology observed in this study.

### Methodological issues

Some methodological issues have to be considered. Dividing a group by a median split could be criticised due to the artificial allocation of patients to a certain group, who would otherwise be described on a continuum. In this study the highest SANS score of the LSS group was 16, whereas the lowest SANS score of the HSS group was 24. This yielded a clear differentiation of schizophrenic patients with low and high negative symptoms.

The chosen neuropsychological tests had to exist in a German version thereby limiting the range of possible assessment procedures. Nevertheless only those tests were taken that are profoundly validated and broadly accepted as neuropsychological diagnostic tools. The neuropsychological tests were administered by a trained and experienced neuropsychologist, guaranteeing an adherent administration. The volumetric method used is sound and has been described in a number of other publications [59-61]. However, according to our measurement protocol we did not separate gray from white matter and thus we cannot commend on whether there might have been a volume reduction of one of these compartments in the different groups.

The patient assessment was done by an experienced team of clinicians on a ward specialized on the treatment of chronic schizophrenia and the psychopathology was validated and quantified using internationally accepted psychometric tools.

### Summary

In summary in this study we present data of a carefully diagnosed sample of patients with schizophrenia and control subjects. Following broad and parallel clinical, psychometric and MRI-based volumetric assessment of temporal and frontal lobe areas we did not identify group overall volumetric differences in frontal or temporal brain volumes. High scores of negative symptoms were categorically and dimensionally related in particular to verbal memory deficits rather than to dysexecutive dysfunction. Therefore the main finding of this study is, that from a functional point of view and in spite of absence of clear macroscopic volumetric brain differences negative symptoms are more related to left temporal brain properties (verbal memory deficits) than to frontal lobe dysfunction (dysexecutive symptoms). Further research should specifically adress this important research question and should possibly test the putative role of temporal glutamatergic dysfunction and its relationship to negative symptoms.

### Ethic committee

AZ: 23–7532.22-11/1 L TvE, Ethik-Kommission der Albert-Ludwigs-Universität, Engelbergerstr. 21, 79106 Freiburg.
